# RF coil design for accurate parallel imaging on ^13^C MRSI using ^23^Na sensitivity profiles

**DOI:** 10.1002/mrm.29259

**Published:** 2022-05-30

**Authors:** Juan D. Sanchez‐Heredia, Rie B. Olin, James T. Grist, Wenjun Wang, Nikolaj Bøgh, Vitaliy Zhurbenko, Esben S. Hansen, Rolf F. Schulte, Damian Tyler, Christoffer Laustsen, Jan H. Ardenkjær‐Larsen

**Affiliations:** ^1^ Department of Health Technology Technical University of Denmark Kgs. Lyngby Denmark; ^2^ Department of Physiology, Anatomy and Genetics University of Oxford Oxford UK; ^3^ Oxford Centre for Clinical Magnetic Resonance Research University of Oxford Oxford UK; ^4^ Department of Radiology Oxford University Hospitals Trust Oxford UK; ^5^ Institute of Cancer and Genomic Sciences University of Birmingham Birmingham UK; ^6^ National Space Institute Technical University of Denmark Kgs. Lyngby Denmark; ^7^ MR Research Centre, Department of Clinical Medicine Aarhus University Aarhus Denmark; ^8^ GE Healthcare Munich Germany

**Keywords:** flexible RF coil, hyperpolarization, parallel Imaging, X‐Nuclei MRI

## Abstract

**Purpose:**

To develop a coil‐based method to obtain accurate sensitivity profiles in ^13^C MRI at 3T from the endogenous ^23^Na. An eight‐channel array is designed for ^13^C MR acquisitions. As application examples, the array is used for two‐fold accelerated acquisitions of both hyperpolarized ^13^C metabolic imaging of pig kidneys and the human brain.

**Methods:**

A flexible coil array was tuned optimally for ^13^C at 3T (32.1 MHz), with the coil coupling coefficients matched to be nearly identical at the resonance frequency of ^23^Na (33.8 MHz). This is done by enforcing a high decoupling (obtained through highly mismatched preamplifiers) and adjusting the coupling frequency response. The SNR performance is compared to reference coils.

**Results:**

The measured sensitivity profiles on a phantom showed high spatial similarity for ^13^C and ^23^Na resonances, with average noise correlation of 9 and 11%, respectively. For acceleration factors 2, 3, and 4, the obtained maximum g‐factors were 1.0, 1.1, and 2.6, respectively. The ^23^Na profiles obtained in vivo could be used successfully to perform two‐fold acceleration of hyperpolarized ^13^C 3D acquisitions of both pig kidneys and a healthy human brain.

**Conclusion:**

A receive array has been developed in such a way that the ^13^C sensitivity profiles could be accurately obtained from measurements at the ^23^Na frequency. This technique facilitates accelerated acquisitions for hyperpolarized ^13^C imaging. The SNR performance obtained at the ^13^C frequency, compares well to other state‐of‐the‐art coils for the same purpose, showing slightly better superficial and central SNR.

## 1 INTRODUCTION

Parallel imaging^1^ has become a standard strategy to accelerate acquisitions and improve encoding efficiency of MR imaging. Standard parallel imaging requires prior information of the receive coil sensitivities to correct for the geometric aliasing artifacts created as data are sampled below the Nyquist rate. The coil sensitivity profiles can be obtained prior to the actual scan (pre‐calibration), or obtained from a fully sampled center of k‐space (auto‐calibration).^2^

In hyperpolarized MRI, imaging time is scarce due to the rapid polarization decay of the hyperpolarized sample. Parallel imaging is then an appealing approach, since in this case the SNR penalty is ideally only proportional to the coil g‐factor (for multi‐shot imaging, with flip angles adjusted to spend the same amount of magnetization^3^). However, the separate acquisition of the sensitivity profiles is not a viable option, since the thermal (non‐hyperpolarized) signal available is prohibitively low. This is especially true for in vivo hyperpolarized ^13^C imaging,^4^ due to the low natural abundance of the ^13^C isotope (1.11%).

In the hyperpolarized ^13^C case, several approaches have been proposed to estimate the coil profiles. One solution is to acquire them in advance from phantom measurements and co‐register these spatially to the in vivo measurements in the reconstruction.^5^, ^6^ Another proposed solution is to add fiducial markers to the coils, such that the position of each element can be calculated and allow for numerical generation of the sensitivity profiles.^7^, ^8^ Both these methods are possible at the low frequency of ^13^C (32.1 MHz at 3T), due to the minor changes on B_1_ field distribution introduced by the sample, which allows to approximate the profiles using Biot‐Savart law (quasi‐static approximation) for the field calculation. However, accurate co‐registration of the individual coil elements from phantom measurements is challenging for flexible arrays, where the conformation of the array is not fixed.

The auto‐calibrated approach has also been applied to hyperpolarized ^13^C imaging.^9^, ^10^ However, this approach does not always perform well in the presence of Gibbs ringing and edge artifacts, which can be a problem for hyperpolarized ^13^C imaging. This is due to the inhomogeneous spatial distribution of the hyperpolarized tracer, with both highly vascularized regions and regions with low metabolic activity, which often creates images with high‐contrast between adjacent voxels.

Finally, a calibrationless method, which does not require estimation of the coil profiles also exists and has been applied to hyperpolarized ^13^C imaging.^11^ When coil profiles cannot be estimated reliably, this method provides a good alternative to acceleration using multi‐channel coil arrays. The method, however, requires a pseudo‐random sampling pattern similar to compressed sensing and is, therefore, not applicable with more standard k‐space trajectories.

To overcome these limitations, a hardware‐based method to obtain highly accurate ^13^C sensitivity profiles is proposed, taking advantage of the proximity in Larmor frequency of the ^23^Na nuclei (1.7 MHz difference at 3T). Endogenous ^23^Na is abundant enough in biological tissue to be imaged with relatively high resolutions within the same scan session as the hyperpolarized ^13^C experiment. It also shows a rather uniform distribution for different tissues, which avoids Gibbs ringing and edge artifacts.^12^ Additionally, obtaining the ^13^C sensitivity profiles during the same hyperpolarized ^13^C scan session, facilitates the integration of parallel imaging into the clinical workflow of the MRI scanner.

The idea in this work was to design a ^13^C receive array (with SNR response optimal at the ^13^C frequency), with a coupling matrix tailored such that the coupling levels were nearly identical at ^13^C and ^23^Na. In this way, the sensitivity profiles are expected to be similar. Some work has already been done to estimate the transmit parameters of the ^13^C scans from the ^23^Na naturally present in biological tissue^13^, ^14^ using commercially available volume coils.

However, for ^13^C multichannel arrays, the situation can be very different. The low loading that the small surface coils experience means that individual coils have a high quality factor (Q‐factor), which increases the coupling between next neighboring coils. This coupling can then be minimized through preamplifier decoupling,^15^ but preamplifier decoupling is also narrowband in frequency for high‐Q coils. Therefore, the similarity of sensitivity profiles between ^13^C and ^23^Na depends directly on what is the exact level of preamplifier decoupling obtained at these two frequencies. For this reason, in a typical ^13^C array, a direct attempt to use the ^23^Na profiles for ^13^C reconstructions will most likely result in suboptimal reconstruction (as shown inRef. [13] for a commercial eight‐channel array).

The design proposed here relies on creating a high level of preamplifier decoupling, which can be obtained by matching the low noise amplifier (LNA) to a higher impedance than the noise optimal, in a controlled way.^16^ Then, its frequency response can be tuned to the intermediate frequency between ^13^C and ^23^Na, such that the coupling levels will still be low (and equal) at both frequencies. As a proof of principle, a flexible eight‐channel array implementing this method is designed and evaluated.

## 2 METHODS

### 2.1 Flexible coil design

In a rigid array high decoupling levels can be achieved through geometric overlap and mismatched preamplifiers.^15^, ^17^ Geometric decoupling is less effective in flexible arrays since coupling coefficients are different for different array conformations. At high frequencies (128 MHz and above), self‐resonant flexible coils are feasible,^18^, ^19^, ^20^ where some sort of transmission line structure is used. Such designs allow some freedom to create magnetic field distributions different from those created by simple loop coils, ultimately allowing reduced mutual coupling based purely on the geometry of the individual coils. At low frequencies, these methods are less practical, since they require very large dimensions of the individual coils. Also, the loading introduced by the biological sample is lower, meaning that any extra loss introduced by the coil materials and electronics has a notable effect on the final coil performance. Therefore, copper loops tuned with high‐Q ceramic capacitors are hard to outperform at these low frequencies.

Based on this rationale, this work follows a different approach for the design of a flexible array, with the use of standard flexible copper wire, tuned using lumped elements. In order to achieve the required decoupling, a high mismatch between the coil and preamplifier is created, as described in Ref. [16], where the coils are matched to a higher impedance (200 Ω, for the sample‐loaded coils) than the optimum for the preamplifier (50 Ω). In this way, a controlled noise penalty is accepted by not matching the coils to the optimal noise impedance of the LNA (WMA32C, WanTCom, USA). This matching enables a level of decoupling above 30 dB (S_21_ < −30 dB), which ultimately allows the flexible array to perform well when conforming to different subjects.

The essential design feature of the proposed array is that the level of decoupling provided by the mismatched preamplifiers should be as similar as possible for the two frequencies of interest. The Noise Figure (NF) of the LNA is 1.3 dB when matched to 200 Ω, compared to 0.7 dB at the optimal impedance of 50 Ω (data obtained from a private communication with the manufacturer, and confirmed experimentally).

Active decoupling from the transmitter was implemented following the low noise circuit design described in Ref. [21], which provided a level of decoupling above 45 dB (S_21_ < −45 dB). Its frequency response was also tuned to be the same at the ^13^C and ^23^Na frequencies, such that any residual B_1_^+^ distortion due to imperfect detuning of the receive coils would be the same for both frequencies. At this point, the noise correlation matrices of an initial four‐channel array were measured, with and without this modification on the preamplifier decoupling response, in order to confirm that the decoupling after the modification was sufficient (see Supporting Information Figure S1, which is available online).

The SNR variation as a function of frequency was measured for one of the array elements by exciting an RF tone through a pickup loop fixed at a distance from the coil element, with the measured spectrum recorded by a spectrum analyzer (PSA E4440A, Keysight, CA, USA). The excited tone was swept over frequency, and the SNR measured as the ratio of the recorded spectrum, divided by the average of a noise spectrum.

**FIGURE 1:**
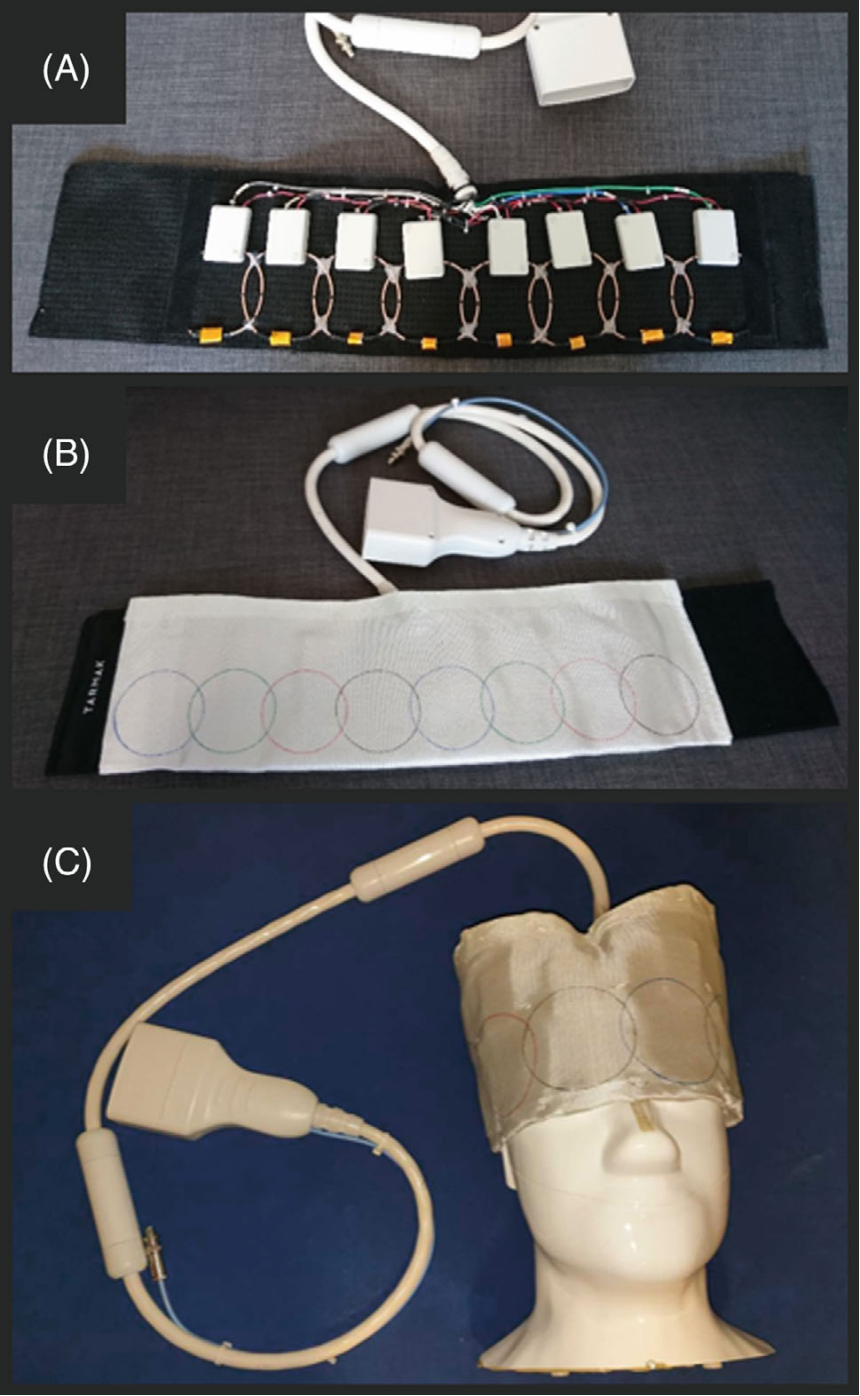
Fabricated flexible eight‐channel ^13^C receive array (f = 32.1 MHz): with the eight‐elements visible sewn into a flexible cloth (A), covered with a flame‐retardant protective cloth (B), and wrapped over the head‐size phantom (C) used for imaging. For each coil element, all the electronic components (matching network, active decoupling, LNA) are integrated into one PCB, and protected with an ABS plastic enclosure (60 × 35 × 15 mm)

The final coil array was made with eight loops of 85 mm diameter each. Each loop was built with standard flexible copper coax (RG‐316), where the outer jacket was used to create the conductive loop. Each individual loop included a trap circuit, which creates a high impedance at the ^1^H frequency, therefore allowing anatomical ^1^H imaging in the presence of the ^13^C array. The trap was made by including a 22 nH inductor (B07T, Coilcraft, USA) in the conductive loop, in parallel with a ceramic capacitor (E‐Series, Johanson Technology, USA). The measured unloaded‐to‐loaded Q‐ratio for the individual elements was Q_U_/Q_L_ = 220/80 when loaded with a human head. The inductance of the individual elements was estimated to be 245 nH, leading to an estimate of equivalent losses of 225 mΩ for the coil, and 394 mΩ for the sample. The coil loss was measured including the solder joints, and the tuning, matching, and decoupling circuitry. The fabricated array is shown in Figure 1.

### 2.2 Array performance: SNR and noise correlation

All MR experiments in this study were performed using a 3T GE MR750 MRI system (GE Healthcare, Milwaukee, USA). The performance of the array was evaluated through MRSI measurements (CSI, FOV = 36 × 36 × 15 cm^3^, matrix size = 24 × 24, slice‐selective pulse [1.8 ms, minimum‐phase], TE = 1.43 ms, flip angle = 70°). The measurements were performed on a Specific Anthropomorphic Mannequin (SAM) phantom filled with ethylene glycol doped with 17 g/L of NaCl (291 mmoL/L ^23^Na concentration) to emulate tissue loading.^22^ The ^13^C measurement was acquired with a TR of 1 s (total acquisition time = 9 min 36 s), while the ^23^Na was acquired with a TR of 219 ms and eight averages (total acquisition time 16 min 40 s). The CSI datasets for each of the two different nuclei were filtered with a 20 Hz line‐broadening filter and the separate coil images were reconstructed based on the first in‐phase time point across the acquired FIDs. Coil images were then normalized to their sum‐of‐squares combined images to remove transmit coil effects and masked to remove the background noise. By normalizing by the combined image, magnitude effects resulting from transmit inhomogeneity cancel out. Masking was done to increase visual clarity. Noise correlation matrices were calculated based on the last 200 points of the raw FIDs. The flip angle calibration was performed using the Bloch‐Siegert shift, as described in Ref. [23].

Finally, the ^13^C SNR was compared with a transmit‐receive birdcage volume coil (RAPID Biomedical, Rimpar, Germany) and a rigid eight‐channel ^13^C receive coil (GE Healthcare, Milwaukee, USA), following the QA protocol described in Ref. [22]. The birdcage is double tuned (^1^H‐^13^C), has an inner diameter of 255 mm and length of 170 mm, while the rigid array is made of rectangular loops of 50 × 100 mm^2^ size. Both coils are designed for human head imaging and are representative of the state‐of‐the‐art of ^13^C coils for brain imaging at 3T. The two paddles of the rigid eight‐channel array were placed directly attached to both sides (left–right) of the head phantom, therefore, placed as close as possible to the phantom.

Measurements done with receive‐only coils were performed using a dedicated ^13^C transmit coil of the clamshell type (RAPID Biomedical, Rimpar, Germany). The electromagnetic design of this coil is based on a Helmholtz pair. This design provides a reasonably similar B_1_^+^ distribution across the frequency range covering the ^13^C and ^23^Na frequencies, as shown in Ref. 13].

### 2.3 Parallel imaging MR experiments

#### 2.3.1 In vitro: Phantom

Parallel imaging reconstruction of ^13^C data using ^23^Na sensitivity profiles based on SENSE^1^ was first verified in phantom experiments, based on retrospectively undersampled data using the SAM phantom described in the previous section.

Data were acquired with a Cartesian sequence, a 2D ^13^C GRE with FOV = 28 × 28 cm^2^, matrix size = 48 × 48, slice thickness = 3 cm, TR = 1000 ms, flip angle = 70°, spectral‐spatial excitation (22 ms pulse length), TE = 9 ms, and total scan time = 32 min.^24^ The dataset was retrospectively undersampled by removing lines in k‐space uniformly, with acceleration rates R = 2, 3, and 4. Coil g‐factors were calculated for each acceleration case.

The ^23^Na data for coil sensitivity mapping were acquired using the same 2D GRE sequence with FOV = 26.6 × 26.6 cm^2^ (5% smaller due to the ^13^C‐^23^Na frequency difference), matrix size = 48 × 48, slice thickness = 2.85 cm, TR = 22.6 ms, TE = 1.43 ms, slice‐selective pulse (1.8 ms, minimum‐phase), total scan time = 21 min 42 s. The flip angle used during this measurement is estimated to be around 20°, because the transmit coil used is not tuned for ^23^Na, and its efficiency at that frequency is very low. SENSE reconstruction requires low T_1_‐weighting; therefore, a relatively long TR was chosen to minimize this effect.

Data were reconstructed in MATLAB R2019b. SENSE reconstruction and g‐factor calculation were done using the code made available by Michael S. Hansen.^25^ Sensitivity maps were estimated from the ^23^Na data by first normalizing the reconstructed multi‐channel images with the sum‐of‐squares combined image and then performing edge‐preserving smoothing using the built‐in MATLAB function “imguidedfilter.” Edge‐preserving smoothing was performed separately for real and imaginary parts of the image data with the default degree of smoothing and a window size set relative to the image size (10% of the number of pixels).

A Cartesian acquisition was used at this part of the study (despite its known inefficiency for ^23^Na), to avoid sequence related artifacts and obtain coil profiles comparable to the Cartesian ^13^C acquisition. In this way an accurate comparison of the ^13^C and ^23^Na coil profiles could be made, with minimal bias from the differences in sequence parameters.

#### 2.3.2 In vivo: Pig kidney and human brain

A 3D ^13^C blipped stack‐of‐spirals (R = 2) sequence^6^ was designed in two versions for ^13^C MR imaging of pig kidneys and human brain, after hyperpolarized [1‐^13^C]pyruvate injection. The sequences were designed with different resolution for pyruvate and metabolites,^26^ with a 45 ms spiral readout, spectral‐spatial excitation,^24^ and with TE = 9 ms. Other sequence parameters can be found in Table 1. Flip angles on the metabolic products were chosen to maximize the total accumulated signal as described previously.^6^

**TABLE 1:** Acquisition parameters for 3D blipped stack‐of‐spirals for imaging of pig kidneys and human brain, respectively

	Pig kidneys		Human brain	
Acq. parameters	Pyruvate	Products	Pyruvate	Products
FOV (cm^3^)	28 × 28 × 10		28 × 28 × 12	
Matrix size	70 × 70 × 10	35 × 35 × 10	40 × 40 × 8	20 × 20 × 8
Nominal spatial resolution (mm^3^)	4 × 4 × 10	8 × 8 × 10	7 × 7 × 15	14 × 14 × 15
Flip angles (°)	9	34	10	28
TR (ms)	86.7		77	
Frequency acq. order	Pyr‐Lac‐Pyr‐Bic‐Pyr‐Ala		Pyr‐Lac‐Bic‐Ala	
Excitations per volume	5		4	
Temporal resolution^a^ (s)	0.867	2.601		1.232

Abbreviations: Ala, alanine; Bic, bicarbonate; Lac, lactate; Pyr, pyruvate.

^a^ Time between full volume acquisitions at the same frequency.

Prior to in vivo acquisition, the ^13^C blipped stack‐of‐spirals sequences were evaluated in vitro using the SAM phantom with the following parameters: TR = 1000 ms, flip angle = 70°, and total scan time = 25 min (for the kidney sequence) and 20 min (for the brain sequence). For comparison, a fully sampled stack‐of‐spirals adaptation of the kidney sequence was also tested with total scan time = 25 min.

CG‐SENSE parallel imaging reconstruction of the non‐Cartesian blipped stack‐of‐spirals datasets was done as previously described,^6^ including 15 Hz filtering on the spiral readouts and B_0_ off‐resonance correction.^27^ But contrary to previous implementation of the off‐resonance correction method, this study did not require any manual shift of the B_0_ map frequency, since the ^13^C center frequency was set directly based on prior information of the ^1^H to ^13^C‐lactate frequency difference on the scanner.^13^

For the in vivo acquisitions, ^23^Na data for sensitivity mapping were acquired using a 3D cones based ultra short echo time sequence, with FOV = 48 × 48 × 48 cm^3^, matrix size = 120 × 120 × 120, 197 excitations per volume acquisition, TR = 120 ms, TE = 0.5 ms, non‐selective excitation, four averages, and total scan time 1 min 35 s. The ^23^Na data were reconstructed using 3D gridding. Sensitivity maps were estimated using the same approach as for the in vitro data, after matching the FOV and resolution to the ^13^C dataset.

The human experiment was approved by the Danish Medicines Agency and the Ethics Committee of the Central Region of Denmark, while the animal experiments were approved by the Danish Animal Inspectorate. The human experiment was performed with a healthy, male volunteer. The pre‐clinical work was performed on a 40 kg healthy female Danish Landrace pig. The pig was anesthetized using propofol and fentanyl. The eight‐channel array was firmly wrapped at the kidney position and adjusted to be most centered in both kidneys. The cross‐section of the pig was approximately 28 cm on the left–right dimension, and 22 cm on the anterior–posterior dimension. The pig was mechanically ventilated and no respiratory gating was used, since motion artifacts are generally low in the kidneys when using fast read‐out sequences.

For anatomical reference, routine proton images were acquired using the body coil of the scanner. For the pig experiment, proton images were acquired using a multi‐slice axial T_2_ Propeller sequence (TR/TE = 6654/100 ms, flip angle = 142°, FOV = 34 × 34 cm^2^, slice thickness = 4 mm, matrix size 256 × 256). For the human experiment, proton images were acquired using a multi‐slice axial fast spin echo sequence (TR/TE = 900/7 ms, flip angle = 111°, FOV = 25 × 25 cm^2^, slice thickness = 8 mm, matrix size 280 × 280). In addition, B_0_ field maps were acquired at the ^1^H frequency for both in vivo studies using the scanner software IDEAL‐IQ (GE Healthcare); a 3D sequence to estimate parameter maps from a single breath‐hold acquisition (TR/TE = 5/2 ms, flip angle = 3°, FOV = 48 × 48 cm^2^ (pig) 44 × 44 cm^2^ (human), slice thickness = 15 mm, matrix size 128 × 128).

For MRI with hyperpolarized pyruvate, [1‐^13^C]pyruvic acid was polarized using dynamic nuclear polarization (SPINlab, GE Healthcare) with AH111501 as an electron paramagnetic agent (EPA), usually yielding >40% polarization.^28^ The sample was dissolved with water for injection and buffered to physiological temperature, acidity, and osmolarity. For the human experiment, the pyruvate was filtered to remove the EPA and quality controlled. The hyperpolarized [1‐^13^C]pyruvate (250 mM) was injected at a dose of 0.43 mL/kg and chased by 20 mL of saline. Imaging was started just after pyruvate injection, before the chaser, to capture the temporal dynamics.

## 3 RESULTS

### 3.1 Array performance

**FIGURE 2:**
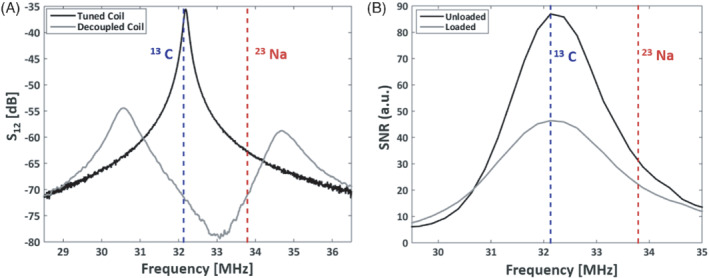
A, Measured S_12_ of a loosely coupled double‐loop probe to one of the coil elements. The frequency response when connected to the preamplifier (“Decoupled Coil”) can be observed and compared to the same coil with the preamplifier disconnected (“Tuned Coil”). This response would normally be tuned to be minimum at the frequency of interests, but in this case it has been tuned to be similar at the ^13^C and ^23^Na frequencies. B, Frequency dependence of the SNR, measured on one of the array elements, with the coil unloaded and loaded

The measured preamplifier decoupling response over frequency for one of the individual elements of the array is shown in Figure 2A. The difference between the optimal decoupling (obtained around 33 MHz) and the decoupling at ^13^C and ^23^Na frequencies is 6–7 dB. This is the level of coupling sacrificed with this design compared to an array optimized for ^13^C. A typical frequency response for one of the coil elements is shown in Figure 2B, which shows that the best SNR performance is obtained at the ^13^C frequency. For a sample loaded coil, the SNR performance at the ^23^Na using this LNA and coil matching, is about half of that at the ^13^C frequency.

**FIGURE 3:**
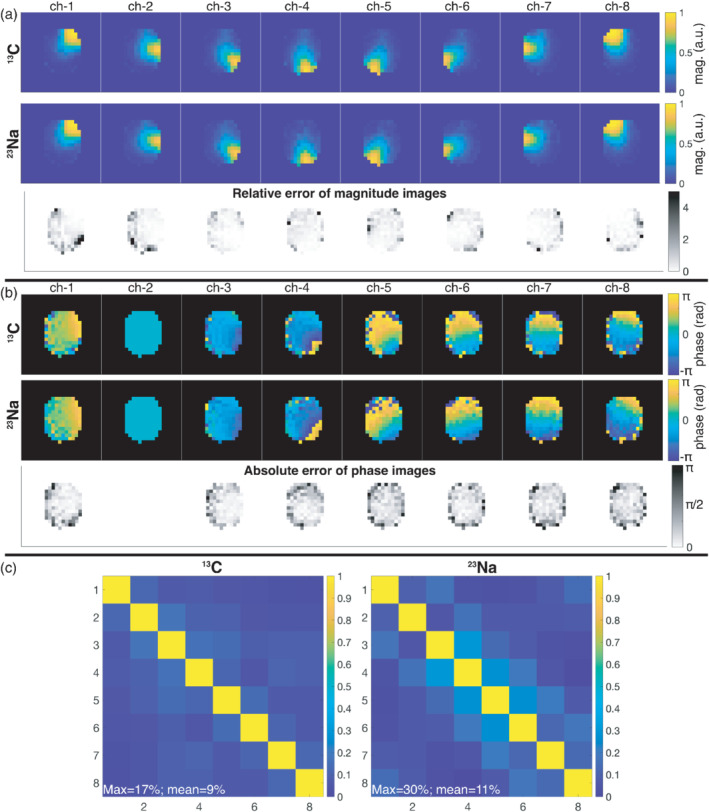
Single‐slice measured coil profiles of the individual array channels for ^13^C and ^23^Na. A, Magnitude images and their relative error. B, Phase images and their absolute error (referenced to the phase of channel 2). C, ^13^C and ^23^Na noise correlation matrices. The noise covariances (normalized to the maximum variance) for ^13^C were: 0.75, 0.75, 0.83, 0.99, 0.80, 0.66, 0.91, 1.00. For ^23^Na, the (normalized) noise covariance were: 0.83, 0.65, 0.77, 0.94, 0.86, 0.60, 0.95, 1.00

In Figure 3A,B, the single‐slice coil profiles are shown measured with the SAM phantom for both ^13^C and ^23^Na frequencies. The relative error of the magnitude images was calculated as the absolute difference divided by the ^13^C magnitude images. Both the relative error of the magnitude images (Figure 3A) and the absolute error of the phase images (Figure 3B) are generally low, especially for the regions close to the coil loops with high SNR. The highest errors are found at the edges opposite the loops, which also correspond to low‐SNR regions where the profile measurements were prone to more errors. Figure 3C shows the noise correlation matrices. Note that, in loaded coils (in the presence of the sample), noise correlation is not a pure metric of coil decoupling. This is because part of the noise correlation between individual coils comes from the fact that nearby coils see the same noise from the sample (e.g., overlapped region on neighboring coils). In any case, the measured values are in general low, and showing good agreement of the average correlation levels between ^13^C and ^23^Na (9% and 11%, respectively).

**FIGURE 4:**
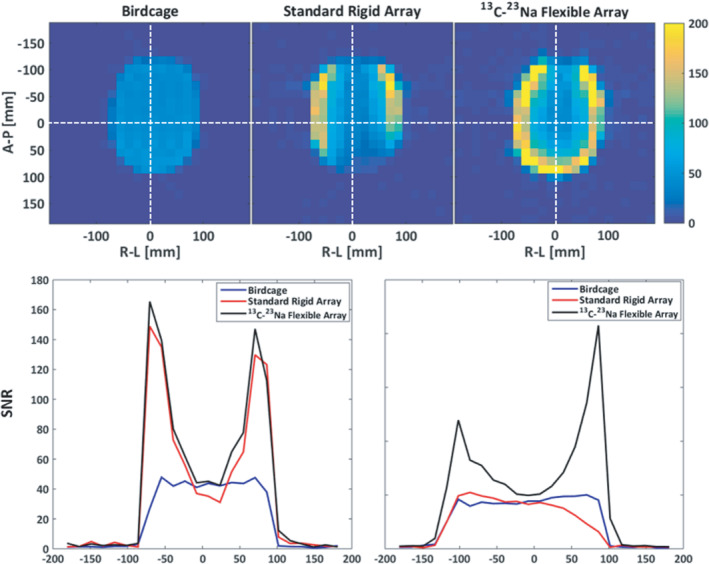
Measured SNR of the fabricated eight‐channel flexible array, compared to a volume coil (of the birdcage type), and to a standard rigid ^13^C eight‐channel array (with two movable paddles of four‐channels each). More information about the reference coils is available in Ref. [22]

Figure 4 shows the ^13^C SNR level of the array compared to the state‐of‐the‐art volume coil and rigid 8‐channel paddle coil. The SNR was measured as described in Ref. [22] over a slice of 20 mm placed at the center of the coil array in the axial plane. The SNR performance from the flexible array compares well to the traditional array, showing slightly better superficial and central SNR. This is most likely due to the slightly increased distance between coil and sample for the rigid array (due to its thicker casing), and the tighter fitting that the flexible array can provide. The comparison to the birdcage volume coil shows that the central SNR is very similar for both coils (≈ 50), with the superficial SNR being three to four times higher for the array.

### 3.2 Parallel imaging performance: Phantom

**FIGURE 5:**
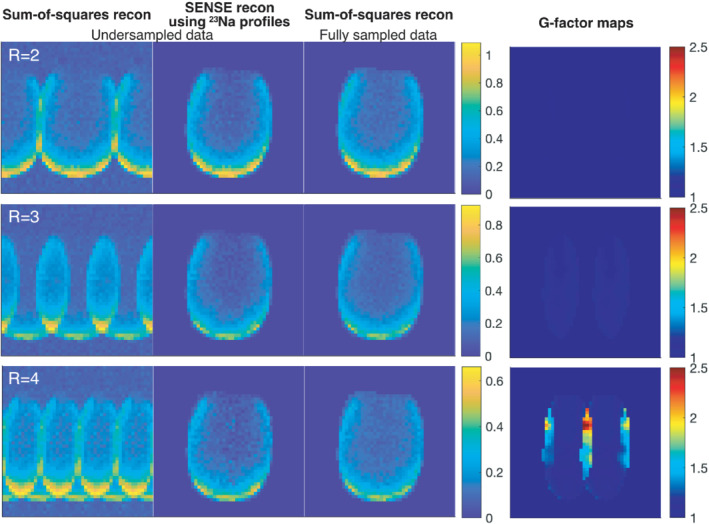
Phantom results with sum‐of‐squares and SENSE reconstructions of the retrospectively undersampled ^13^C data compared to the fully sampled ^13^C data. The images show an axial slice of the head‐shaped SAM phantom in arbitrary units. G‐factor maps are also shown

The results from the in vitro study, demonstrating a SENSE reconstruction of ^13^C data using ^23^Na acquired sensitivity profiles with the same coil array, are shown in Figure 5 for different accelerations rates (R = 2, 3, and 4). The SENSE reconstructions of the undersampled data are compared to the fully sampled sum‐of‐squares reconstruction. After retrospective undersampling, the total scan time amounts to 16 min for R = 2, 11 min for R = 3, and 8 min for R = 4. To approximately match the scan time for the fully sampled data in the comparison, averages were removed resulting in a total scan time of 16 min for the comparison to R = 2 and R = 3, and a total scan time of 8 min for comparison to R = 4.

The sum‐of‐squares reconstructions of the fully sampled data and the SENSE reconstructions of the undersampled data show minimal differences for acceleration rates R = 2 and R = 3. This is also reflected in the associated g‐factor maps, with g‐factors equal to 1 for R = 2 and close to 1 for R = 3, meaning none or minimal noise amplification in the parallel imaging reconstruction. For acceleration rate R = 4, substantial noise amplification is seen in the top‐center of the phantom with g‐factors >2.

**FIGURE 6:**
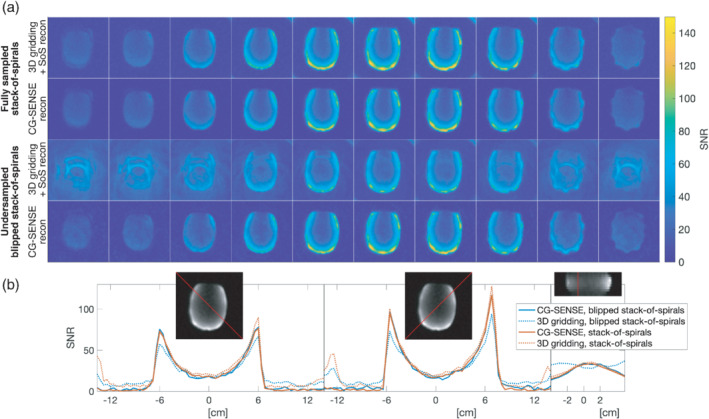
SNR of phantom data acquired using the 3D blipped stack‐of‐spirals and a fully sampled 3D stack‐of‐spirals (10 axial slices shown from left to right). A, From top to bottom: Fully sampled stack‐of‐spirals, first reconstructed using 3D gridding with sum‐of‐squares coil combination, second with CG‐SENSE reconstruction using the ^23^Na acquired coil profiles. Then blipped stack‐of‐spirals, first reconstructed using 3D gridding with sum‐of‐squares coil combination, second with CG‐SENSE reconstruction using the ^23^Na acquired coil profiles. B, SNR profiles for three different cross‐sections. Left and middle: for the two diagonal cross‐sections for a central axial slice. Right: for a superior–inferior cross‐section, referenced to the sagittal plane

Figure 6 shows the high‐resolution SNR images from the phantom test of the variable resolution blipped stack‐of‐spirals ^13^C sequence designed for the pig kidney imaging, compared to a fully sampled stack‐of‐spirals. The CG‐SENSE reconstruction with ^23^Na sensitivity maps is able to remove the majority of the aliasing artifacts seen in the sum‐of‐squares reconstruction in the undersampled dataset while retaining the SNR level. This correlates well with the close to unity g‐factor for the R = 2 accelerated GRE in Figure 5. Notice, however, the superficial “spike‐pattern” artifacts seen in the last two slices in both the fully sampled and undersampled images. These are attributed to B_0_ distortions caused by some of the electronics on the coil (see Supporting Information Figure S1). Due to the very tight fit to the subject, materials and components disturbing the magnetic field slightly, can introduce visible superficial artifacts. With the thin shell of the SAM phantom (2 mm), and hereby short distance from coil to signal, these images represent an extreme case of these artifacts. The artifacts were, therefore, not considered a concern for the in vivo experiments, but highlight the need to carefully choose the position of the electronic boards in flexible arrays.

### 3.3 Parallel imaging performance: In vivo pig kidney and human brain

**FIGURE 7:**
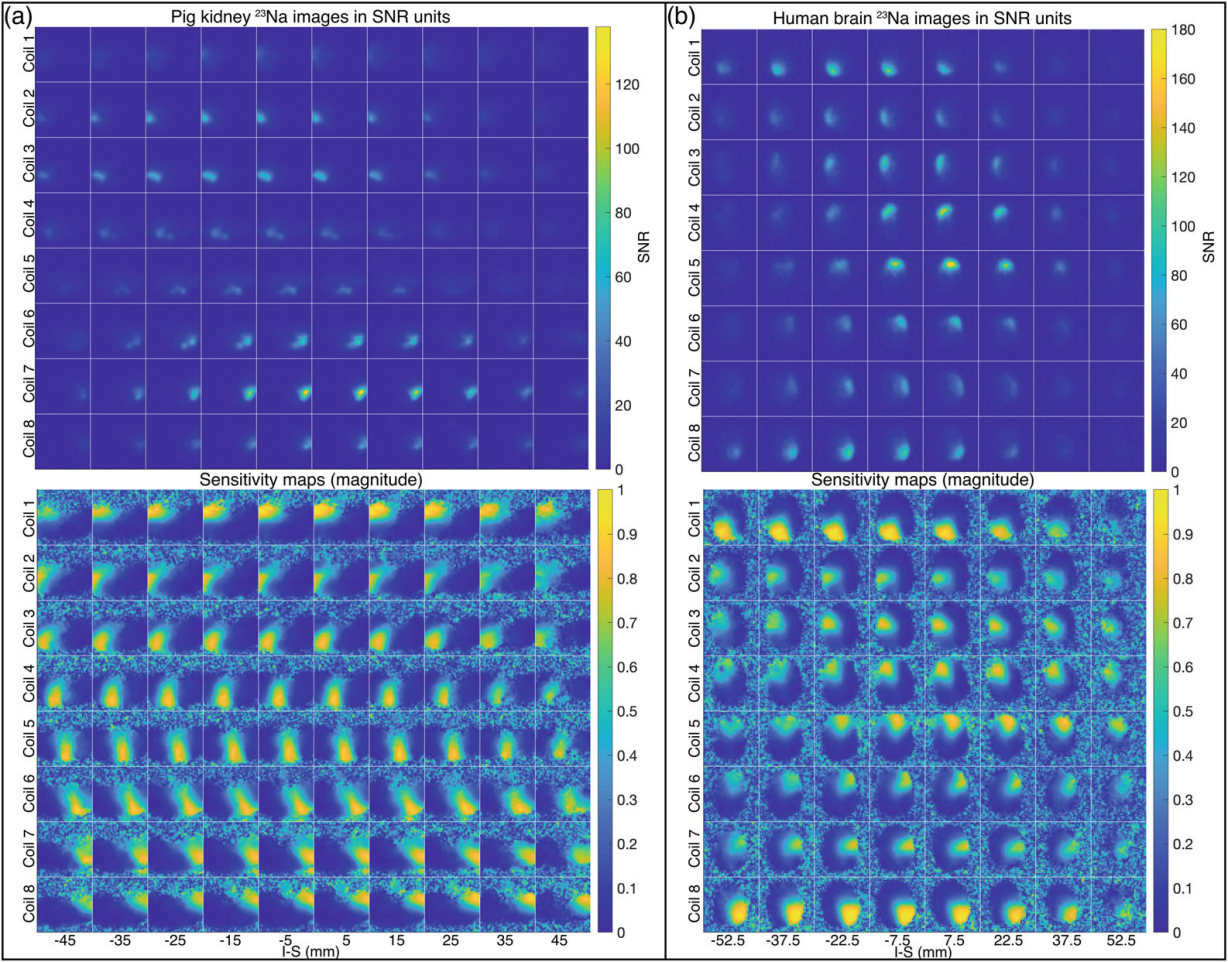
3D ^23^Na SNR images (top) and final magnitude coil sensitivity maps (bottom): for the abdomen of the healthy pig (A), and for the head of the healthy human volunteer (B). The images are shown for axial slices from left to right and coil channels from top to bottom

Finally, the performance of the array was evaluated in vivo in animal and in human. First, the 3D cones ^23^Na data were acquired prior to the hyperpolarization experiments. The reconstructed ^23^Na images after matching the FOV and resolution to the ^13^C pyruvate data are shown in Figure 7 (as SNR maps) for both in vivo studies together with the magnitude of the final processed coil sensitivity maps used for reconstruction of the ^13^C data. The phase images of the coil sensitivity maps are shown in the Supporting Information Figure S1.

The SNR levels are different across the different slices, with a maximum SNR at the central slices and much lower SNR levels at the peripheral slices. This is partly due to the large FOV in the inferior–superior direction (120 mm for the pig and 100 mm for the human study), relative to the size of the coil elements (Ø = 85 mm). From the final sensitivity maps, however, it can be seen that the SNR levels of the 3D ^23^Na images for both the abdomen of the pig and the head of the human are sufficient to clearly delimitate the individual coil positions and field distribution across the full volume, despite signal variations due to both anatomy and coil geometry.

**FIGURE 8:**
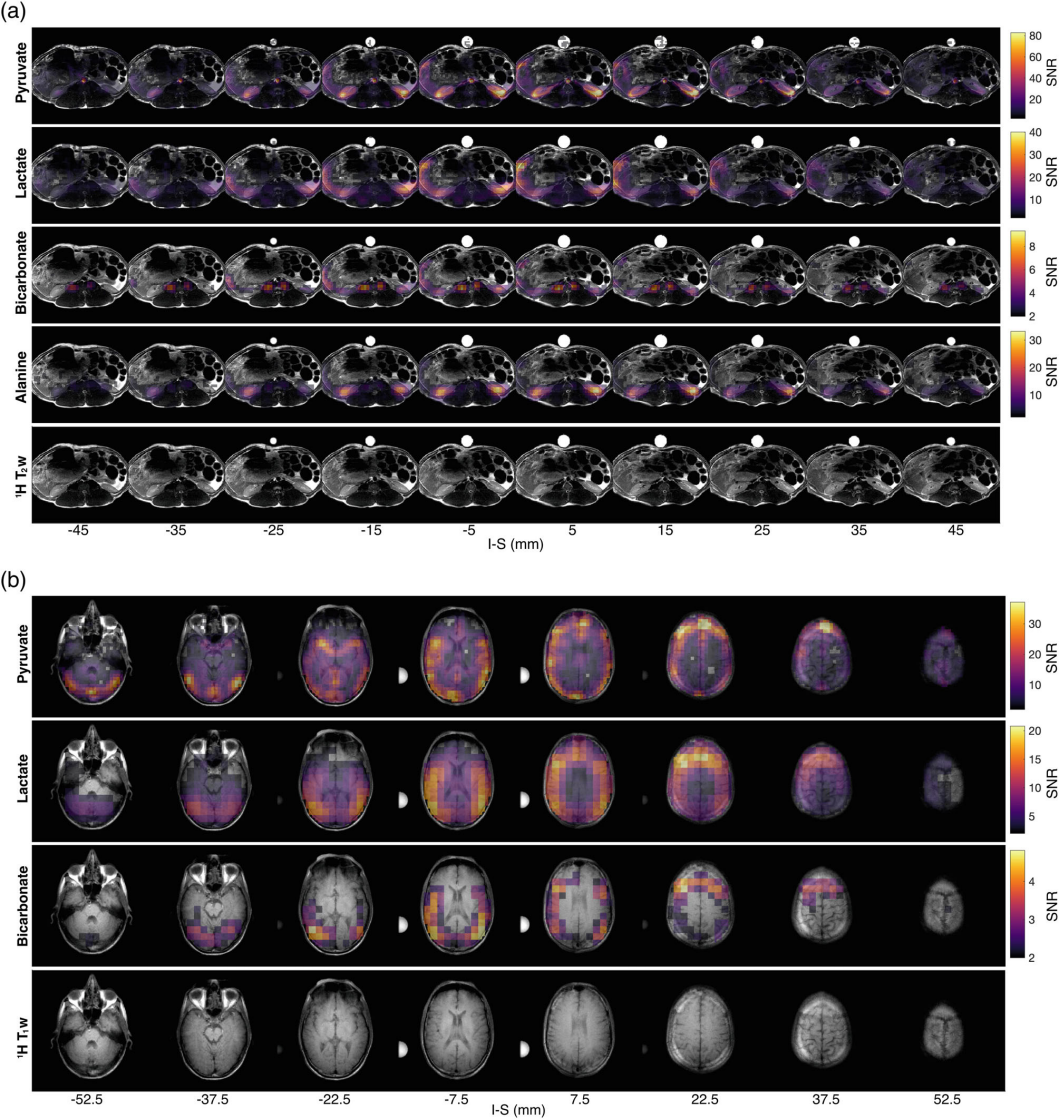
In vivo accelerated (R = 2) blipped stack‐of‐spirals ^13^C MR imaging of pig kidneys and human brain following hyperpolarized [1‐^13^C] pyruvate injection and CG‐SENSE reconstruction using the ^23^Na coil profiles shown in Figure 7. A, Pig kidney metabolic maps across axial slices shown from left to right (top to bottom: pyruvate, lactate, bicarbonate, alanine), with ^1^H T_1_‐weighted images as anatomical reference. B, Human brain metabolic maps across axial slices shown from left to right for (top to bottom: pyruvate, lactate, bicarbonate), with ^1^H T_1_‐weighted images as anatomical reference. The reconstructions include off‐resonance correction. The metabolic maps are shown summed over time. Both ^1^H and pyruvate images were windowed to increase contrast. Pyruvate images were windowed to 60% of the maximum signal. All metabolite images were masked with respect to ^1^H image signal and to SNR levels higher than 2

Figure 8 shows the time‐averaged metabolic maps after hyperpolarized [1‐^13^C] pyruvate injection for both in vivo experiments. The images are shown as transparent overlays on anatomical ^1^H images. Metabolite‐only images are shown in Supporting Information Figure S1. Typical metabolic activity is observed both in the healthy pig kidneys and in the healthy human brain. The brain metabolism distribution and the image quality are comparable to previously reported experiments with similar conditions.^26^ Alanine images are not shown for the brain, due to insufficient signal.

Supporting Information Videos S1 and S2 show the metabolic dynamics in animations of the pig kidney and human brain data, respectively. Supporting Information Figures S1 show more images from the brain data and provides transparency to the reconstruction pipeline. Supporting Information Figure S1 shows the CG‐SENSE‐reconstructed metabolic maps compared to a 3D gridded, sum‐of‐squares reconstruction without off‐resonance correction, similar to the phantom images in Figure 6. Supporting Information Figure S1 shows the pyruvate and lactate images following different reconstruction procedures. Supporting Information Figure S1 shows the mean time curves.*

## 4 DISCUSSION

This work investigates the possibility of introducing a hardware modification into an eight‐channel flexible array dedicated for ^13^C MR, such that its individual sensitivity profiles can be accurately obtained from biologically abundant ^23^Na. It is shown that for an array where the decoupling between individual elements is very high (S_21_ < −30 dB), a sacrifice can be made on the tuning of the preamplifier decoupling response, such that the ^13^C and ^23^Na coupling matrices are similar. The ^13^C SNR level measured with this array shows an adequate performance, comparable to two state‐of‐the‐art coils for ^13^C brain imaging, which have shown good SNR performance in previous studies.^22^, ^29^ In general, this result confirms that the extra coupling accepted at the ^13^C frequency with this method does not imply a remarkable effect on the final ^13^C SNR performance, for the FOV of a human head. It should also be taken into account that the FOV coverage of the coils is different across the z‐axis; therefore, the SNR performance at the central slice does not provide all the information needed to categorically evaluate a coil. In this case, it is used to have an objective metric to benchmark the SNR performance of the proposed array, since a 20 mm slice thickness is relevant for hyperpolarized ^13^C MRSI.

The in vitro results demonstrate that the ^23^Na coil profiles are sufficient for sensitivity mapping and parallel imaging reconstruction of ^13^C data with acceleration factors up to R = 4 for the given undersampling strategy relative to the eight‐channel coil geometry. Geometry factors for acceleration factors up to R = 3 were insignificant. The SAM phantom has a relatively high ^23^Na concentration (291 mmol/L) compared to in vivo conditions in the brain (gray matter: 36 mmol/L, white matter: 31 mmol/L, cerebrospinal fluid [CSF]: 125 mmol/L).^30^ This, together with the homogenous distributions of both ^13^C and ^23^Na signal, provided an advantageous starting point for testing the coil setup. However, despite the more challenging conditions in vivo, results from both the pig abdomen and human brain show successful parallel imaging reconstruction of undersampled (R = 2) hyperpolarized ^13^C data by means of sensitivity profiles from ^23^Na data obtained with the same coil in the same subject. The results obtained from the human brain are comparable in quality to those reported for similar experiments using arrays with higher channel‐count (e.g., 24‐channel array in Ref. [26]).

Regarding the ^23^Na imaging performance, this study is only a proof of principle, and significant hardware improvements can be undertaken to obtain better ^23^Na SNR, especially regarding transmit coil efficiency. In fact, a better design for this purpose has recently been proposed,^14^ where a large‐FOV four‐rung birdcage could be tuned optimally at the ^23^Na frequency, with a power efficiency only 3 dB lower at the ^13^C frequency.

The results presented here show that it is possible to obtain sufficient ^23^Na SNR from both the pig abdomen and human brain tissue in a reasonable time (1 min 30 s), even with a suboptimal ^23^Na coil setup. However, this may be more challenging for other body parts with smaller ^23^Na concentration, or higher ^23^Na gradients.^31^ For example, there is a 90–95 mmol/L difference between tissue and CSF in the brain.^30^, ^32^ However, as there is no ^13^C signal from pyruvate or its metabolites in CSF (due to no metabolism nor direct feeding from the supplying vessels to the brain), this is unlikely to distort image domain unfolding. For thoracic and abdominal imaging, self‐gating acquisitions have already been successfully used for ^23^Na imaging, to avoid blurring from breathing motion.^33^ Additionally, due to the low frequencies, quasi‐static field approximations can be made to overcome this limitation. Such field approximations can also be used to estimate the absolute sensitivity profiles from the ^23^Na coil images, which would allow for intensity correction of the final metabolic maps. In the current implementation, ^23^Na coil images were normalized to their sum‐of‐squares combined image to cancel out anatomic signal variations before estimating the relative sensitivity profiles. In cases where intensity correction is not needed, for example, for calculating metabolic rate maps, this is the most straightforward and recommended approach.

The conclusions of this study are not general and will not work equally well for any coil geometry and array channel count. For example, bigger coils or higher Q coils do require higher decoupling to prevent performance degradation; therefore, this method might not be suitable for those. In the specific case of flexible arrays, high decoupling is already a need to minimize detuning of the different elements for the changing configurations and matching conditions. Also in these arrays, the high sample loading experienced due to their better anatomical fitting provides a broader frequency response of their SNR performance (as shown in Figure 3), making them less sensitive to detuning due to coupling. This SNR vs. frequency response is dependent on the preamplifier noise response for different input impedances and can, therefore, also potentially be improved, if one designs one's own preamplifier.

It is important to mention that the method proposed here for receive arrays can only be used if the transmit coil used has a sufficient degree of similarity in its B_1_^+^ profiles for the ^13^C and ^23^Na frequencies. In this work, a commercial ^13^C transmit coil is used, where the B_1_^+^ similarity had already been verified.^13^

In summary, with the proposed method parallel imaging of hyperpolarized ^13^C can be integrated and automated, with less sources of error compared to pre‐calibration methods that need accurate co‐registration of the profiles and regular coil control to assure that the calibrated profiles match the current state of the coil. Furthermore, this method also allows parallel imaging with flexible coils, which can provide increased SNR through tight patient‐specific fitting.

## 5 CONCLUSIONS

This work demonstrates a method to obtain accurate sensitivity profiles for hyperpolarized ^13^C imaging from the ^23^Na naturally present in biological samples, provided that the receive hardware is specifically designed for that purpose. A flexible eight‐channel receive array for ^13^C at 3T (32.1 MHz) has been developed based on this method, and its parallel imaging performance has been evaluated in vitro and in vivo in both a hyperpolarized ^13^C pig kidney and human brain experiment, showing successful results.

This method can be used to obtain accurate ^13^C sensitivity profiles in cases where the low natural abundance of ^13^C makes it impossible, facilitating the use of accelerated sequences for hyperpolarized ^13^C experiments.

### ACKNOWLEDMENTS

This work has been partly funded by the Danish Council for Independent Research (DFF – 4005‐00531), the Danish National Research Foundation (DNRF124), Lundbeck Foundation (R272‐2017‐4023 & R278‐2018‐620), and the European Research Council (ERC‐856432, HyperQ). Dr. James Grist was funded by the Oxford BHF Centre of Research Excellence by grant code (RE/18/3/34214), and by a European Commission grant (858 149, AlternativesToGd). Prof. Damian Tyler was funded by a British Heart Foundation Senior Research Fellowship (FS/19/18/34252).

## Supporting information


**FIGURE S1** (A) Sensitivity profiles measured for the ^13^C and ^23^Na frequencies with a regular 4‐channel array (with preamplifier decoupling tuned optimally for ^13^C). (B) Similar measurement with the same array modified to have similar decoupling at ^13^C and ^23^Na. (C) Noise correlation matrices measured with the regular array, and (D) with the modified array
**FIGURE S2** Measured B_0_ maps [Hz] for 6 slices of 10 mm across the central part of the head phantom. (A) SAM phantom alone, and (B) with the flexible 8‐channel array placed around it. (B) shows the superficial B_0_ artifacts (pointed with arrows) that the flexible coil creates, especially in the first slices (which are closer to the electronic boards)
**FIGURE S3** Phase images of the final in vivo ^23^Na‐calibrated coil sensitivity maps: (A) for the abdomen of the healthy pig, and in (B) for the head of the healthy human volunteer
**FIGURE S4** In vivo accelerated (R = 2) blipped stack‐of‐spirals ^13^C MR imaging of (A) pig kidneys and (B) human brain, both following hyperpolarized [1‐^13^C] pyruvate injection and CG‐SENSE reconstruction using the ^23^Na coil profiles shown in Figure 7. The reconstructions include off‐resonance correction. The metabolic maps are shown summed over time, and pyruvate images are windowed to 60% of the maximum signal to improve contrast
**FIGURE S5** (A) Human brain ^13^C hyperpolarized images acquired with the blipped stack‐of‐spirals sequence and reconstructed with sum‐of‐squares (SoS) and CG‐SENSE. The CG‐SENSE reconstruction include off‐resonance correction based on the B_0_ map in Hz in (B). Pyruvate images were windowed to 60% of the maximum signal to suppress signal from the superior sagittal sinus
**FIGURE S6** Human brain ^13^C hyperpolarized images for pyruvate (top) and lactate (bottom), summed over time, after different reconstruction procedures. Pyruvate images were windowed to 60% of the maximum signal to suppress signal from the superior sagittal sinus
**FIGURE S7** Mean time curves across the full image volume (after applying ^1^H mask) for the human brain ^13^C hyperpolarized experiment. Dashed lines are the acquired data, solid lines are after smoothing with a generalized moving average filter (Savitzky–Golay) over a span of 10 data points

**VIDEO S1** 3D pig kidney hyperpolarized ^13^C full dynamics after smoothing with a generalized moving average filter (Savitzky–Golay) over a span of 5 data points voxel‐wise. Metabolites are shown in separate rows, scaled to their own maximum at each time point. In the top right corner for each row, this maximum is stated relative to the maximum pyruvate signal

**VIDEO S2** 3D human brain hyperpolarized ^13^C full dynamics after smoothing with a generalized moving average filter (Savitzky–Golay) over a span of 10 data points voxel‐wise. Metabolites are shown in separate rows, scaled to their own maximum at each time point. In the top right corner for each row, this maximum is stated relative to the maximum pyruvate signal

## Footnotes

* We provide access to raw data through Zenodo, https://doi.org/10.5281/zenodo.6359357.
