# Supplementary material for: RF coil design for accurate parallel imaging on 13C MRSI using 23Na sensitivity profiles
Source: Magn Reson Med. 2022 May 30;88(3):1391–405. doi: 10.1002/mrm.29259 (PMC9328386; doi:10.1002/mrm.29259)
Supplement: Supplementary file 1 — FIGURE S1 (A) Sensitivity profiles measured for the 13C and 23Na frequencies with a regular 4‐channel array (with preamplifier decoupling tuned optimally for 13C). (B) Similar measurement with the same array modified to have similar decoupling at 13C and 23Na. (C) Noise correlation matrices measured with the regular array, and (D) with the modified array FIGURE S2 Measured B0 maps [Hz] for 6 slices of 10 mm across the central part of the head phantom. (A) SAM phantom alone, and (B) with the flexible 8‐channel array placed around it. (B) shows the superficial B0 artifacts (pointed with arrows) that the flexible coil creates, especially in the first slices (which are closer to the electronic boards) FIGURE S3 Phase images of the final in vivo 23Na‐calibrated coil sensitivity maps: (A) for the abdomen of the healthy pig, and in (B) for the head of the healthy human volunteer FIGURE S4 In vivo accelerated (R = 2) blipped stack‐of‐spirals 13C MR imaging of (A) pig kidneys and (B) human brain, both following hyperpolarized [1‐13C] pyruvate injection and CG‐SENSE reconstruction using the 23Na coil profiles shown in Figure 7. The reconstructions include off‐resonance correction. The metabolic maps are shown summed over time, and pyruvate images are windowed to 60% of the maximum signal to improve contrast FIGURE S5 (A) Human brain 13C hyperpolarized images acquired with the blipped stack‐of‐spirals sequence and reconstructed with sum‐of‐squares (SoS) and CG‐SENSE. The CG‐SENSE reconstruction include off‐resonance correction based on the B0 map in Hz in (B). Pyruvate images were windowed to 60% of the maximum signal to suppress signal from the superior sagittal sinus FIGURE S6 Human brain 13C hyperpolarized images for pyruvate (top) and lactate (bottom), summed over time, after different reconstruction procedures. Pyruvate images were windowed to 60% of the maximum signal to suppress signal from the superior sagittal sinus FIGURE S7 Mean time curves across the full image vol [file MRM-88-1391-s002.docx]

**Supporting Information**


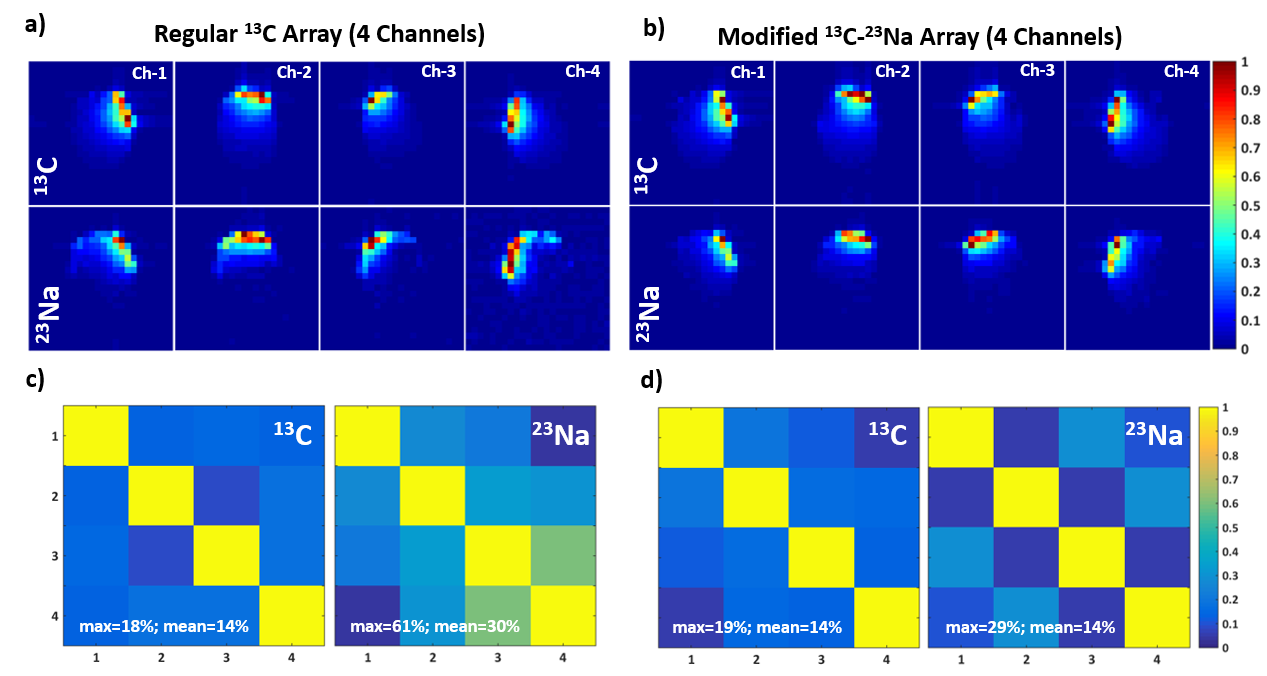


Supporting Information Figure S1. A) Sensitivity profiles measured for the ^13^C and ^23^Na frequencies with a regular 4-channel array (with preamplifier decoupling tuned optimally for ^13^C). B) Similar measurement with the same array modified to have similar decoupling at ^13^C and ^23^Na. C) Noise correlation matrices measured with the regular array, and D) with the modified array.


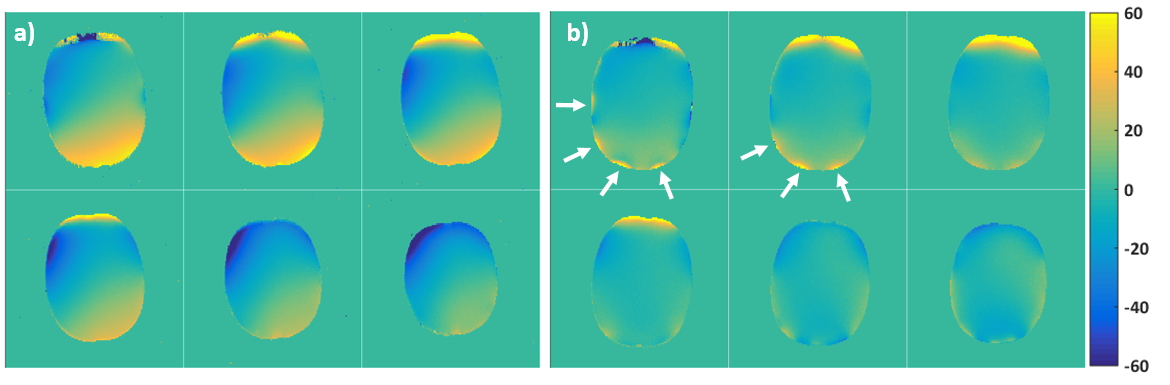


Supporting Information Figure S2. Measured B_0_ maps [Hz] for 6 slices of 10 mm across the central part of the head phantom. A) SAM phantom alone, and B) with the flexible 8-channel array placed around it. B) shows the superficial B_0_ artifacts (pointed with arrows) that the flexible coil creates, especially in the first slices (which are closer to the electronic boards).


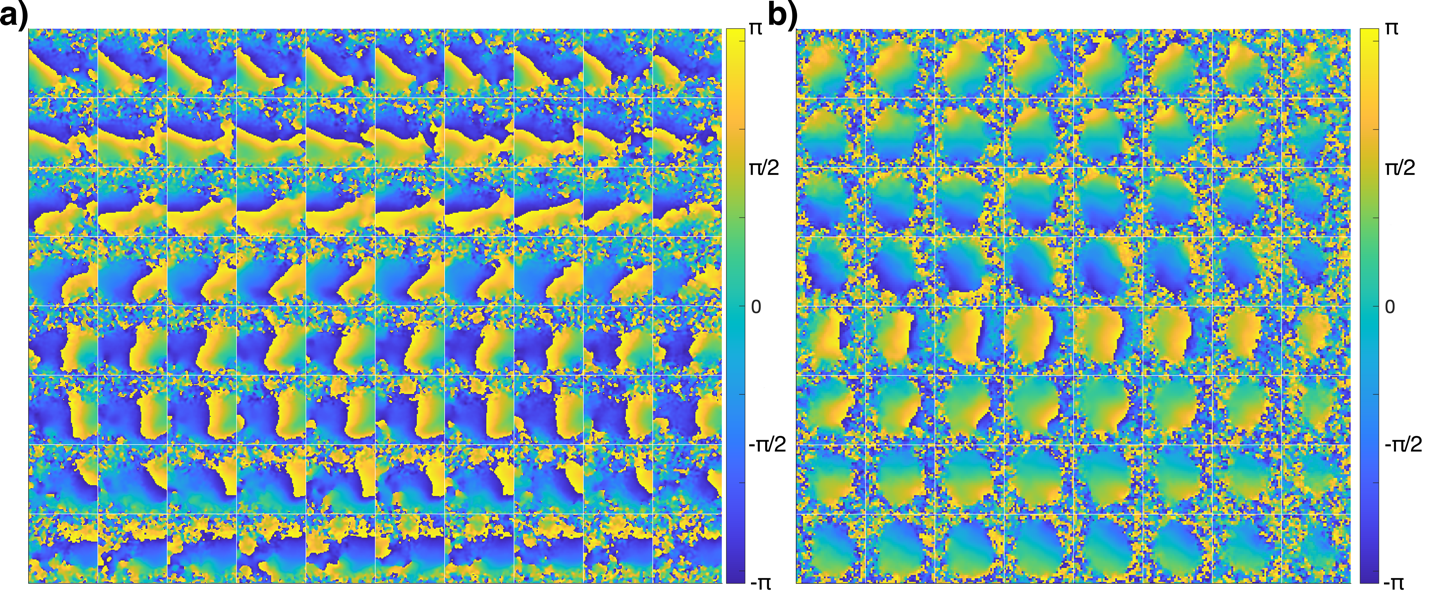


Supporting Information Figure S3. Phase images of the final in vivo ^23^Na-calibrated coil sensitivity maps: a) for the abdomen of the healthy pig, and in b) for the head of the healthy human volunteer.


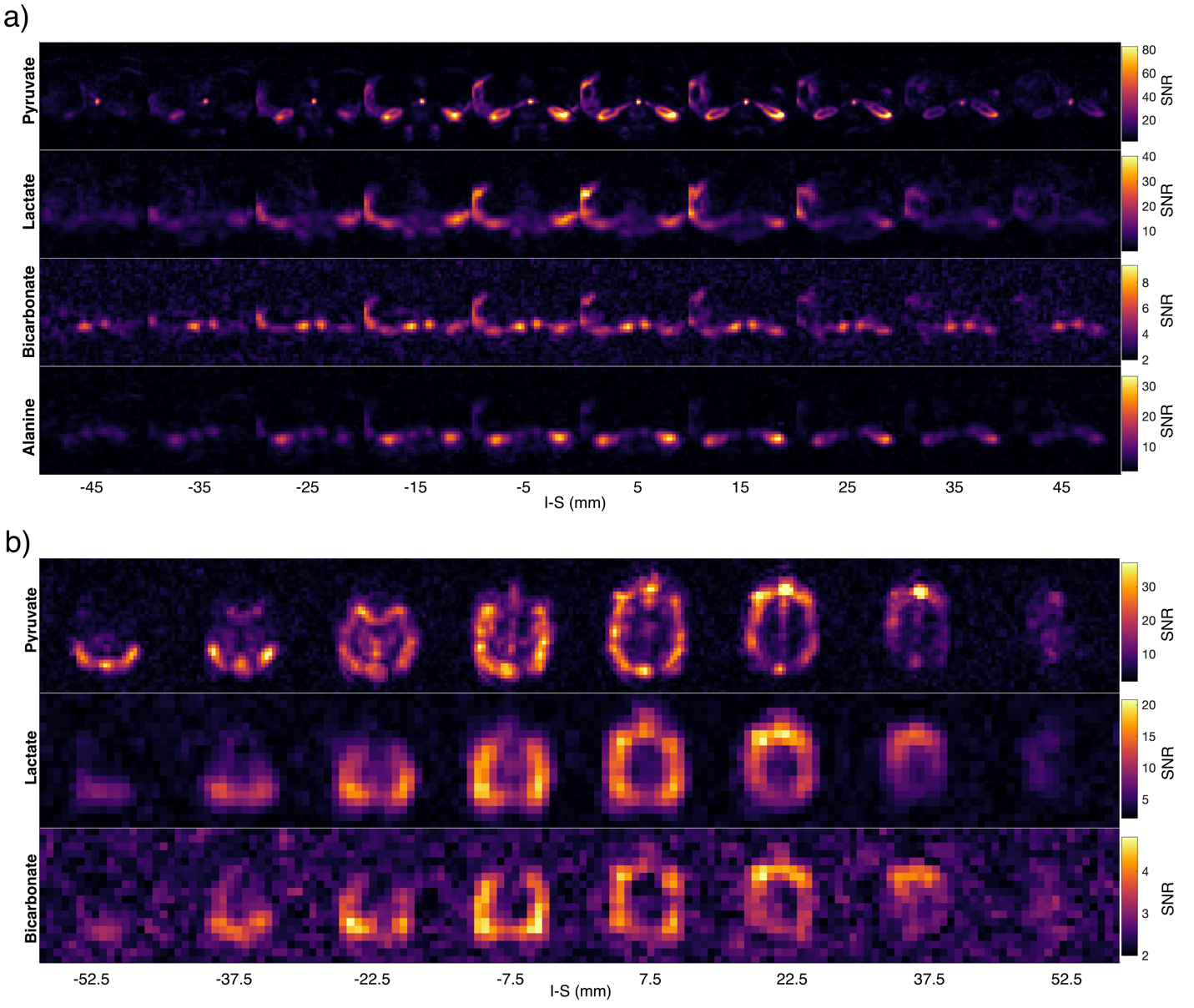


Supporting Information Figure S4. In vivo accelerated (R=2) blipped stack-of-spirals ^13^C MR imaging of a) pig kidneys and b) human brain, both following hyperpolarized [1-^13^C] pyruvate injection and CG-SENSE reconstruction using the ^23^Na coil profiles shown in Fig. 7. The reconstructions include off-resonance correction. The metabolic maps are shown summed over time, and pyruvate images are windowed to 60 % of the maximum signal to improve contrast.


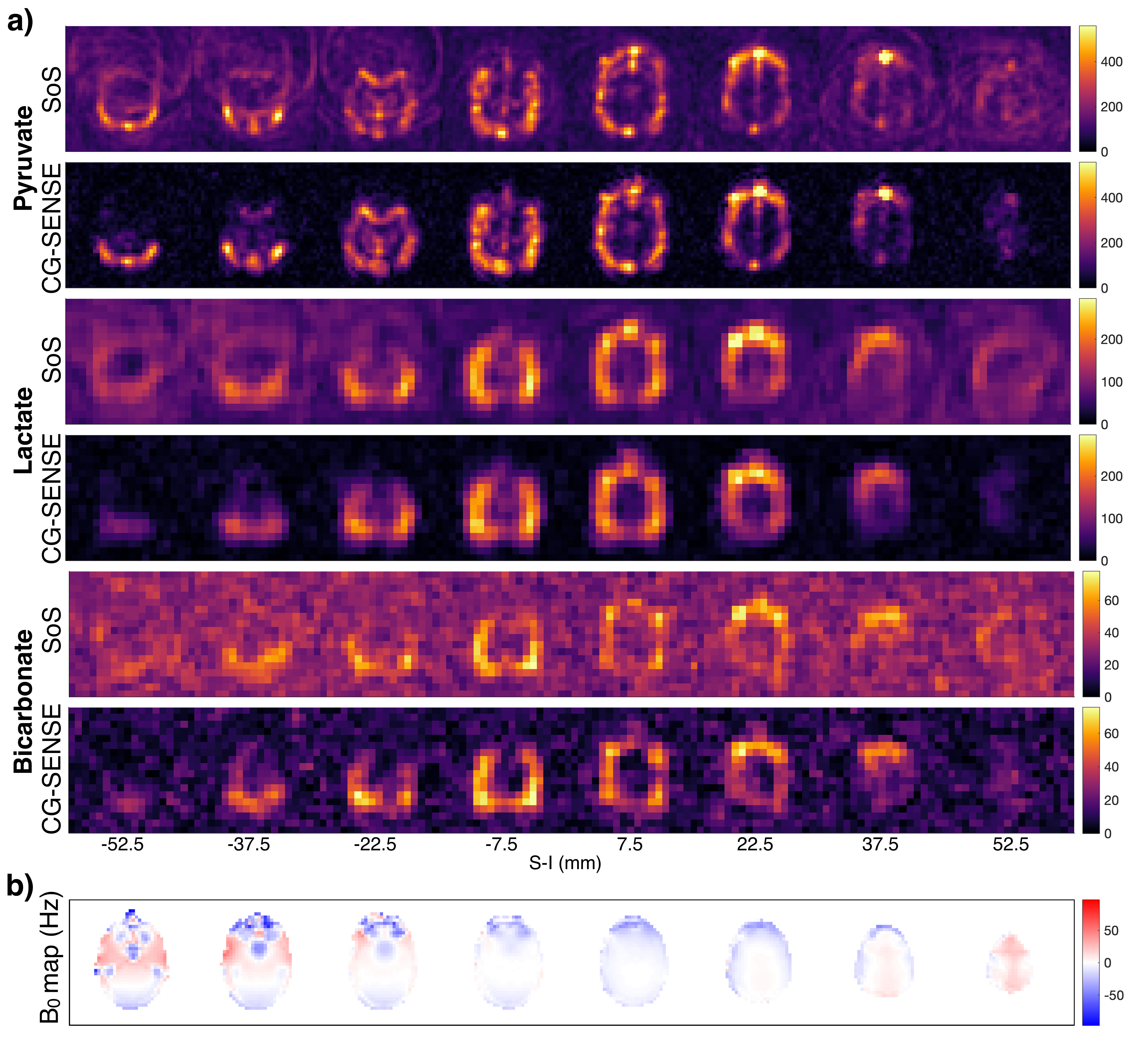


Supporting Information Figure S5. a) Human brain ^13^C hyperpolarized images acquired with the blipped stack-of-spirals sequence and reconstructed with sum-of-squares (SoS) and CG-SENSE. The CG-SENSE reconstruction include off-resonance correction based on the B_0_ map in Hz in b). Pyruvate images were windowed to 60% of the maximum signal to suppress signal from the superior sagittal sinus.


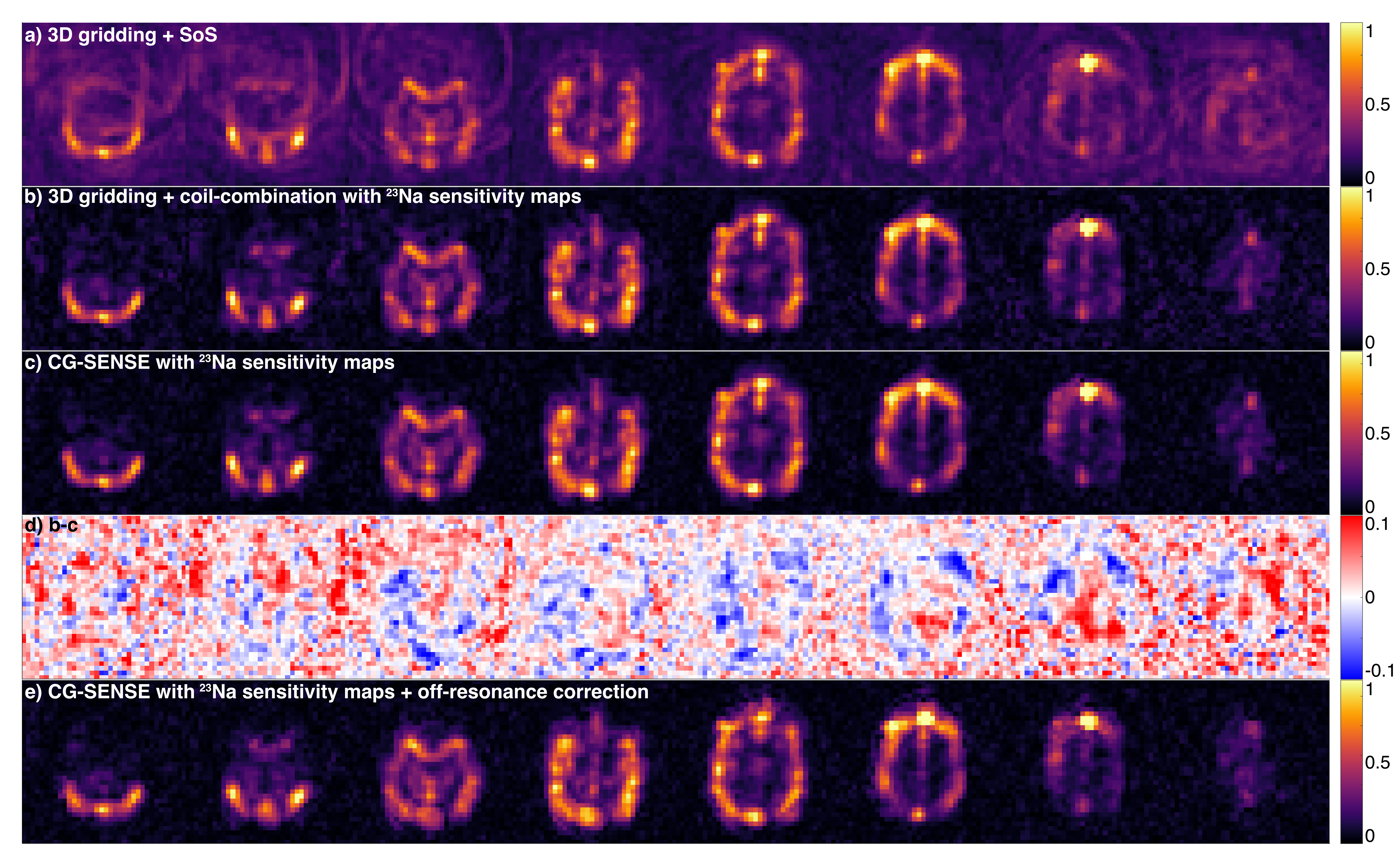

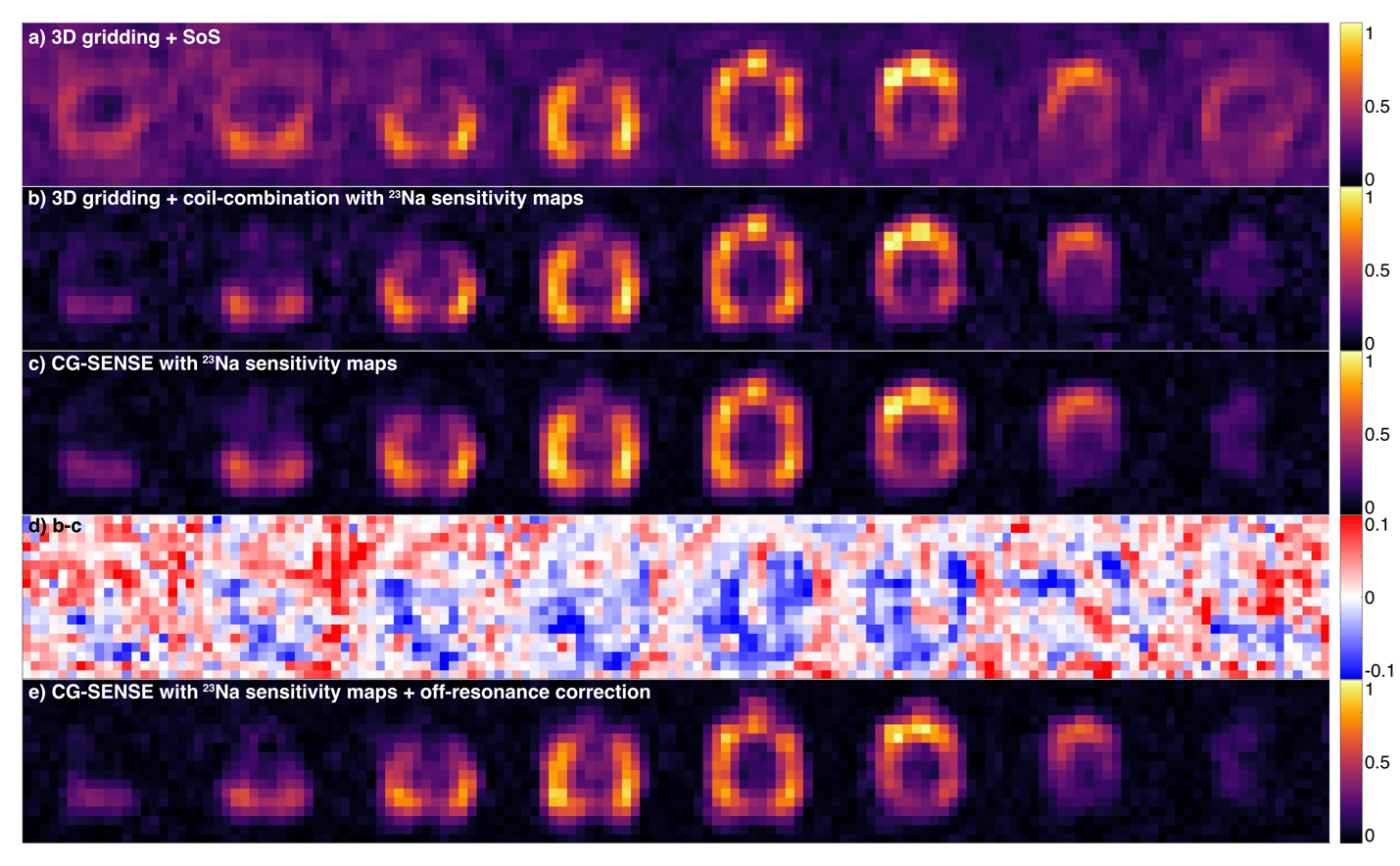


Supporting Information Figure S6. Human brain ^13^C hyperpolarized images for pyruvate (top) and lactate (bottom), summed over time, after different reconstruction procedures. Pyruvate images were windowed to 60% of the maximum signal to suppress signal from the superior sagittal sinus.


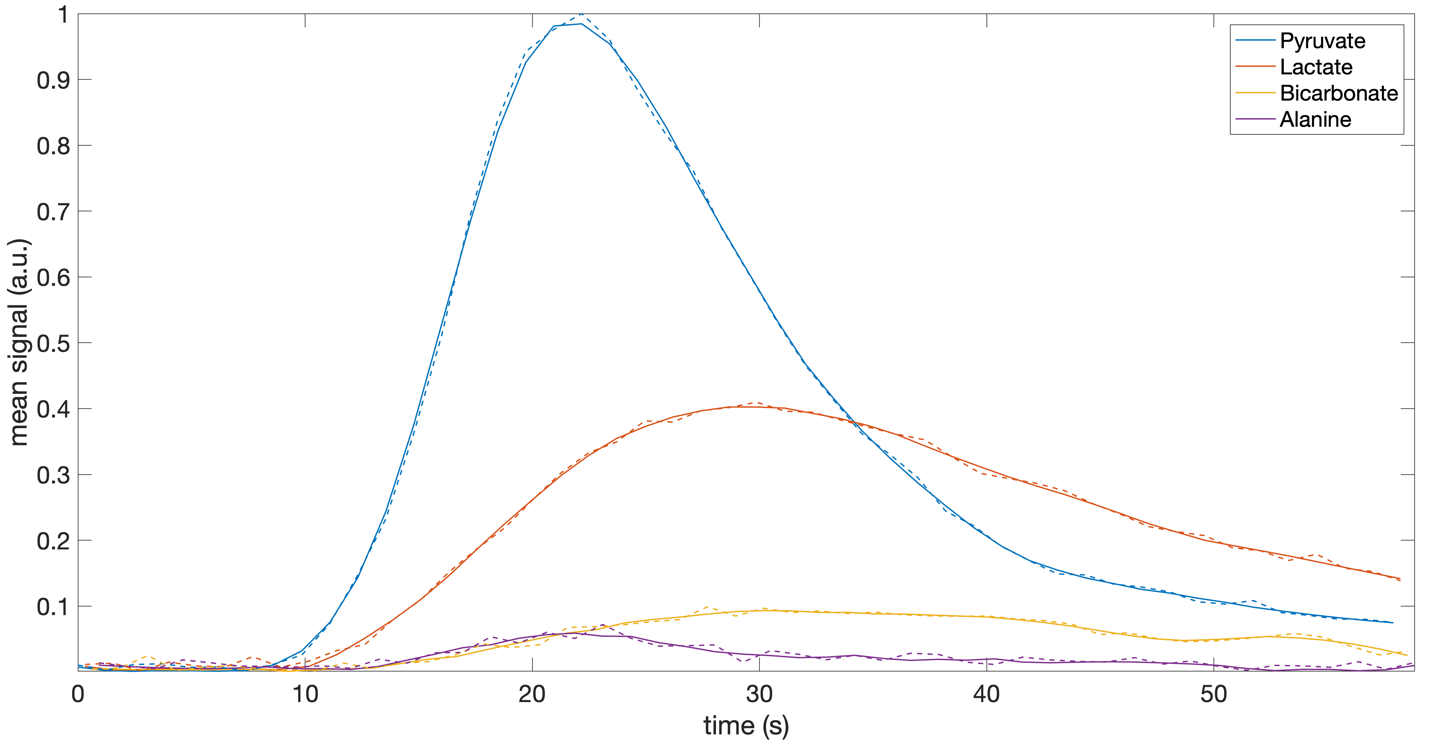


Supporting Information Figure S7. Mean time curves across the full image volume (after applying ^1^H mask) for the human brain ^13^C hyperpolarized experiment. Dashed lines are the acquired data, solid lines are after smoothing with a generalized moving average filter (Savitzky-Golay) over a span of 10 data points.

Supporting Information Video S1. 3D pig kidney hyperpolarized ^13^C full dynamics after smoothing with a generalized moving average filter (Savitzky-Golay) over a span of 5 data points voxel-wise. Metabolites are shown in separate rows, scaled to their own maximum at each time point. In the top right corner for each row, this maximum is stated relative to the maximum pyruvate signal.

Supporting Information Video S2. 3D human brain hyperpolarized ^13^C full dynamics after smoothing with a generalized moving average filter (Savitzky-Golay) over a span of 10 data points voxel-wise. Metabolites are shown in separate rows, scaled to their own maximum at each time point. In the top right corner for each row, this maximum is stated relative to the maximum pyruvate signal.
